# Origins and Neurochemical Characteristics of Porcine Intervertebral Disc Sympathetic Innervation: a Preliminary Report

**DOI:** 10.1007/s12031-017-0956-3

**Published:** 2017-07-31

**Authors:** Monika Barczewska, Judyta Juranek, Joanna Wojtkiewicz

**Affiliations:** 1 Department of Neurology and Neurosurgery, Faculty of Medical Sciences, University of Warmia and Mazury, Olsztyn, Poland; 2 Department of Pathophysiology, Faculty of Medical Sciences, University of Warmia and Mazury in Olsztyn, Ul. Warszawska 30, 10-082 Olsztyn, Poland; 3 Laboratory for Regenerative Medicine, Faculty of Medical Sciences, University of Warmia and Mazury, Olsztyn, Poland; 4 Foundation for the Nerve Cells Regeneration, Olsztyn, Poland

**Keywords:** Intervertebral disc diseases, Retrograde tracer, Pigs, Intervertebral disc sympathetic innervation

## Abstract

Intervertebral disc diseases (IVDDs) form a group of a vertebral column disorders affecting a large number of people worldwide. It is estimated that approximately 30% of individuals at the age of 35 and approximately 90% of individuals at the age of 60 and above will have some form of disc-affecting pathological changes leading to disc herniation, prolapse and degeneration as well as discogenic pain. Here, we aimed to establish the origins and neurochemical characteristics of porcine intervertebral disc sympathetic innervation involved in pain signalling in IVDD patients. Pigs were given an injection of the Ominipaque contrast agent and Fast Blue (FB) retrograde tracer into the L_4_-L_5_ intervertebral disc and euthanized at 2, 1, and 3 months post injection. Following euthanasia, bilateral sympathetic chain ganglia (SChG) Th_13_ to C_1_ were collected. The presence, distribution and neurochemical characteristics of retrogradely labelled SChG neurons were examined. The majority (88.8%) of all FB+ cells were found in the L_3_-L_5_ SChG. Most FB+ neurons stained for dopamine beta hydroxylase (DBH); one-third to one-quarter stained for somatostatin (SOM), neuropeptide Y (NPY) or leu-enkephalin (LENK); and only a few stained for galanin (GAL). Compared with the control, the greatest decline in neurochemical immunostaining was observed 2 weeks post injection, and the lowest decline was noticed 1 month post injection. Our study, for the first time, provides insight into the complex patterns of intervertebral disc sympathetic innervation and suggests that the best time for neurochemical balance restoration therapy would be 1 month post-injury, when the neuronal concentration of all studied substances is close to the initial physiological level, thus providing favourable conditions for successful recovery.

## Introduction

Intervertebral disc diseases (IVDDs) are a group of a vertebral column disorders affecting a large number of people worldwide (Teraguchi et al. 2014). It is estimated that approximately 30% of people at the age of 35 and approximately 90% of people at the age of 60 and above will have some form of disc-affecting pathological changes leading to disc herniation, disc prolapse and overall disc degeneration, which will, in turn, lead to a wide variety of uncomfortable symptoms, such as a painful degenerating disc or, in severe cases, walking difficulties, thus negatively impacting the quality of life of those affected with the disease (Alshami 2015; Teraguchi et al. 2014).

The available small animal models of intervertebral disc diseases are often inadequate and either fail to provide a complex mechanistic explanation of disc degeneration that is observed in human patients or are fairly limited in uncovering new therapeutic approaches to disc disease prevention and treatment (Alini et al. 2008). To circumvent the limitations of small animal models, in recent years, a great deal of attention has been directed towards larger models, such as pigs, which, due to their anatomical and physiological features, more accurately mimic human diseases and reflect the pathological changes underlying many of them in greater detail compared with other animal models. Porcine intervertebral discs are similar in size and structure to human discs, which makes them a very convenient and reliable model for detailed studies of disc deterioration pathogenesis and for testing novel therapeutic approaches to painful disc degeneration in human patients (Busscher et al. 2010).

Sympathetic neurons are thought to contribute to the increased back pain sensation in patients with intervertebral disc degeneration. Studies have shown that so-called “discogenic” pain, which stems from degenerating or injured discs, might be the result of increased sympathetic innervation supplying these discs, contributing to “peripheral sensitization” and increased pain sensation in patients with IVDDs (Peng et al. 2005).

Here, we aim to elucidate the origins and neurochemical characteristics of sympathetic neurons supplying intervertebral discs in large domestic pigs, which are closely physiologically and anatomically similar to humans. The results will provide the groundwork for further preclinical studies of the pathogenesis of disc degeneration and discogenic pain and contribute to clinical experiments on intervertebral disc physiology and pathophysiology in disc disease therapy.

## Materials and Methods

Large White Polish juvenile female pigs, weighing 25–30 kg, were used in the study. The animals were divided into four groups: one control (C) and three experimental (E1–3). All groups were injected with 350 μl of a fluorescent retrograde tracer Fast Blue (FB)/radiologic contrast agent solution (Omnipaque; 300 mg Iohexolum/ml; Takeda Ireland Ltd., Citywest, Republic of Ireland) into the L_4_-L_5_ intervertebral disc. The duration of the experiment was designed based on previous studies, and the following end time points were established: group E1 – 2 weeks post injection; group E2 – 1 month post injection; and group E3 – 3 months post injection. In addition to FB injections, the three experimental groups underwent nucleus pulposus dehydration by one-minute disc laser vaporisation (400 J energy pulse, Dornier Medilas D MultiBeam, Germany), followed by introducing a laser fibre optic probe into the intervertebral space with an epidural anaesthesia needle, which was performed under C-arm fluoroscopy (Siemens Medical Solutions) guidance as previously described (Barczewska et al. 2013). The position of the needle was checked radiologically before depositing the solution into the nucleus pulposus of the studied disc. All animal procedures were performed in accordance with Animal Welfare Guidelines and approved by the local Ethics Committee at the University of Warmia and Mazury, Olsztyn, Poland. All animals were sheltered in the core animal facility at the Faculty of Veterinary Medicine, University of Warmia and Mazury. The care and comfort of each animal was safeguarded in compliance with all pertinent animal welfare laws, regulations, and policies. Each study was planned, conducted and completed with due and acceptable regard for the welfare of the animal.

All animals in one experimental group were euthanized with pentobarbital overdose at their respective end time points and transcardially perfused with a freshly prepared 4% buffered paraformaldehyde. The bilateral sympathetic chain ganglia (SChG) from Th_15_ to S_3_ were collected, cryoprotected and sectioned on a cryostat into 10-μm-thick serial sections for further analysis. Each section was examined for the presence, distribution and diameter of FB-positive SChG neurons under a BX61 fluorescence microscope (Olympus, Poland). The immunofluorescent characterisation of the SChG neurochemical phenotype was performed according to the standard protocol as previously described (Barczewska et al. 2013), and the following primary antibodies were used: dopamine beta hydroxylase (DBH), neuropeptide Y (NPY), galanin (GAL), leu-enkephalin (LENK) and somatostatin (SOM) (Table 1). The omission or replacement of primary antibodies on sections from each tissue sample set was carried out simultaneously with the experimental staining.

**Table 1:** List of primary and secondary antibodies used in the study

Primary antibodies				
Antibody	Code	Host Species	Dilution	Supplier
DβH	MAB 308	mouse	1:1000	Chemicon International Inc., UK; www.chemicon.com
NPY	NA 1233	rabbit	1:10,000	Biomol Research Laboratories Inc., US; www.biomol.com
SOM	8330–0154	rabbit	1:10,000	Biogenesis Inc., UK; www.biogenesis.co.uk
GAL	RIN7153	rabbit	1:10,000	Peninsula Labs, US; see Bachem AG; www.bachem.com
LENK	EA 1149	rabbit	1:10,000	Affinitiy BioReagents Inc., UK; www.bioreagents.com
Secondary antibodies				
Reagent			Dilution	Supplier
Donkey anti-mouse IgG (H + L) FITC conjugated			1:800	715–095-151; Jackson IR Lab, US; www.jacksonimmuno.com
Biotinylated goat anti-rabbit immunoglobulins			1:1000	E0432, DAKO Corporation, US, www.dakousa.com
Biotin conjugated F(ab)’ fragment of affinity purified anti-rabbit IgG (H + L)			1:1000	711–1622, BioTrend, Germany; www.biotrend.com
CY3- conjugated Streptavidin			1:9000	016–160-084, Jackson IR Lab, US; www.jacksonimmuno.com

Statistical analysis was performed using GraphPad Instat (GraphPad, La Jolla, CA, USA). All values are presented as the mean ± standard error (SEM).

## Results

After applying the tracer to the nucleus pulposus of the L4-L5 intervertebral disc, FB+ sensory neurons were found in the bilateral Th_16_-S_2_ SChG. Most FB+ cells were found between the L_2_ and L_6_ SChG, constituting 94.6% of all FB+ cells. The relative distribution of FB-labelled neurons in the collected SChG was as follows: Th_16_–0.4%, L_1_–0.9%, L_2_ 4.5%, L_3_–11.5%, L_4_–63.5%, L_5_–11.8%, L_6_ – 3.3%, S_1_–0.95%, and S_2_–1.1% of all labelled neurons. The average number of FB+ cells observed in the injected disc was approximately 842 cells per animal (ranging from 651 to 1148).

**Fig. 1:**
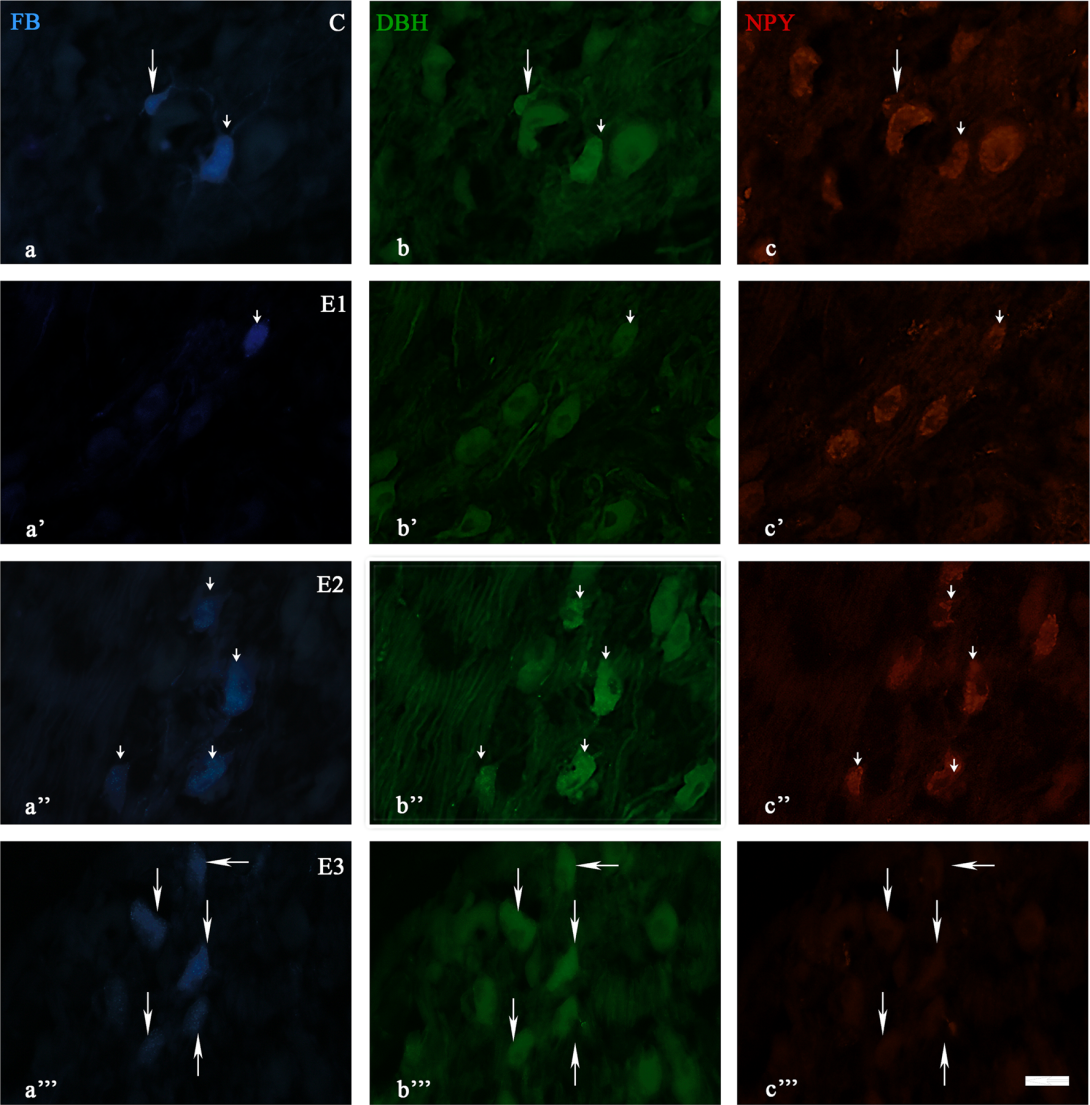
Immunofluorescent images showing DBH and NPY immunofluorescence in SChG neurons of the control (C) and experimental groups (E1, E2, E3) after FB injection into the porcine intervertebral discs. The *small arrow*(s) show(s) the FB-positive neurons containing two examined substances (a-c; a’-c’; a”-c”). The *arrow*(s) show(s) the FB-positive neuron(s) containing DBH (a-c; a”-c”)

**Fig. 2:**
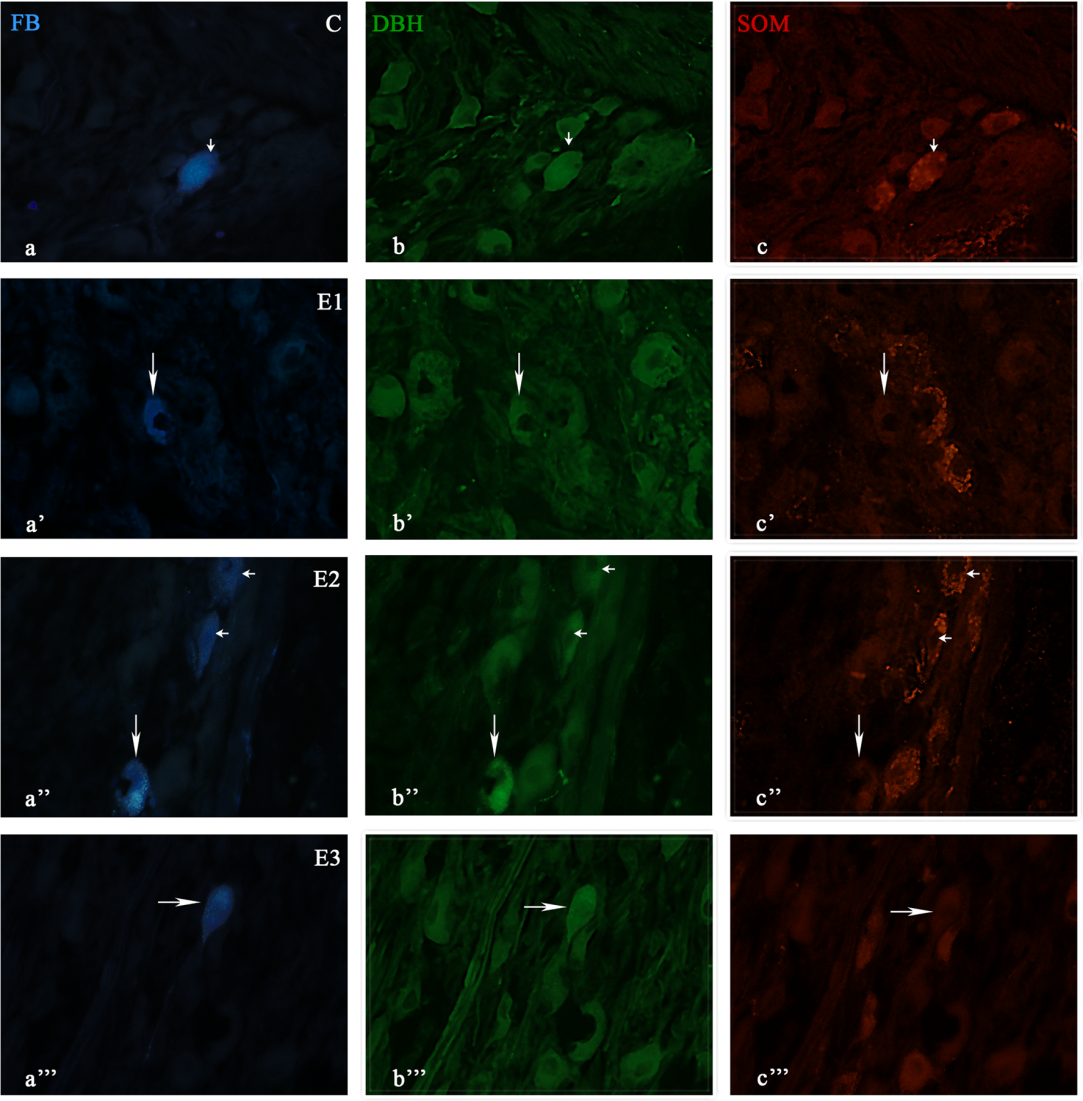
Immunofluorescent images showing DBH and SOM immunofluorescence in SChG neurons of the control (C) and experimental groups (E1, E2, E3) after FB injection into the porcine intervertebral discs. The *small arrow*(s) show(s) the FB-positive neurons containing two examined substances (a-c; a”-c”). The *arrow*(s) show(s) the FB-positive neuron(s) containing DBH (a’-c’; a”-c”, a”’-c”’)

**Fig. 3:**
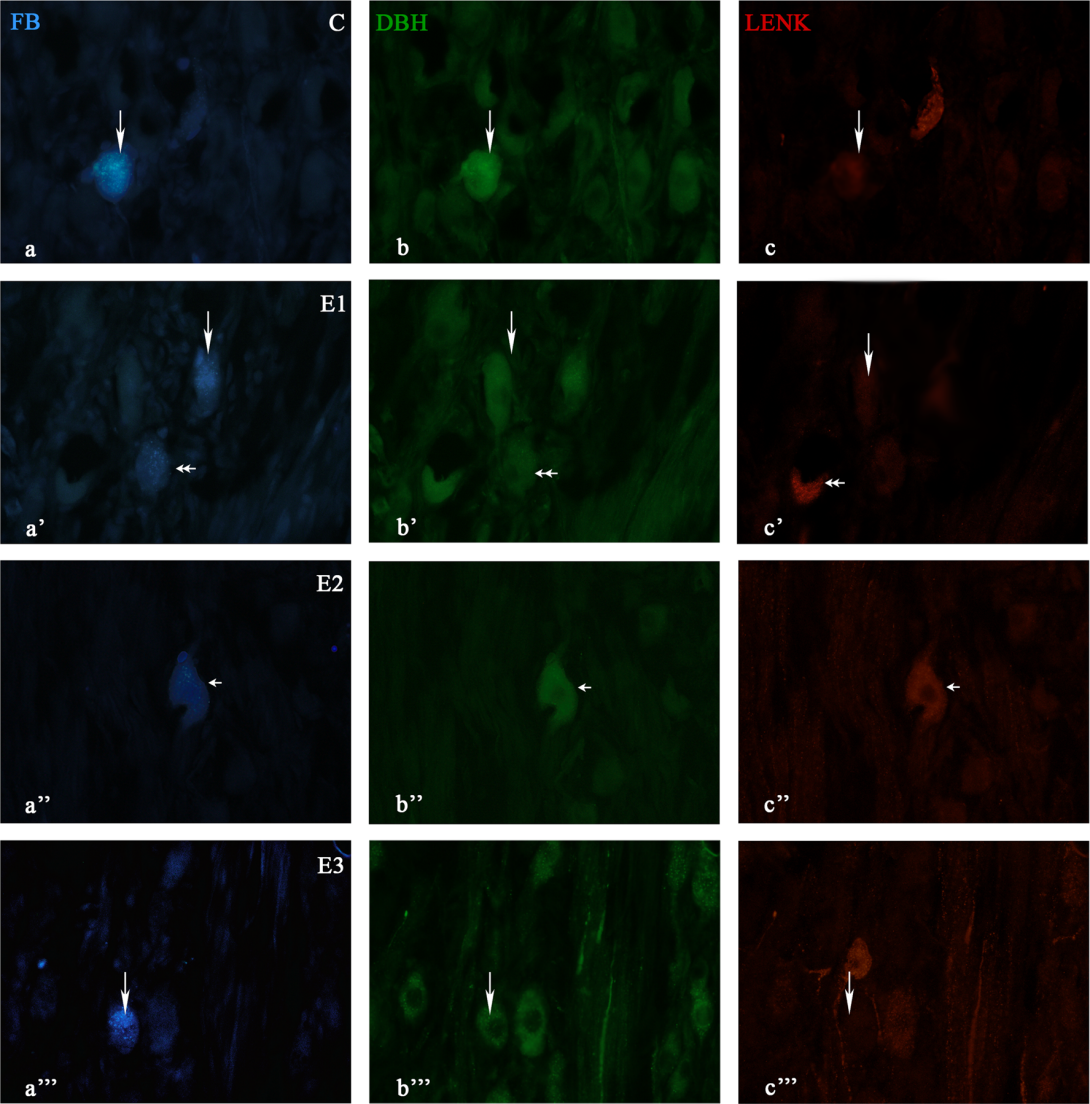
Immunofluorescent images showing DBH and LENK immunofluorescence in SChG neurons of the control (C) and experimental groups (E1, E2, E3) after FB-injection into the porcine intervertebral discs. The *small arrow*(s) show(s) the FB-positive neurons containing two examined substances (a”-c”). The *arrow*(s) show(s) the FB-positive neuron(s) containing DBH (a-c; a’-c’; a”’-c”’). The *double small arrow* indicates LENK (a’-c’)

In all four groups, the majority of all FB+ neurons were positive for DBH. One-third to one-quarter of the FB+ neurons stained for SOM, NPY, or LENK. Finally, only a few of the FB+ neurons stained for GAL (Figs. 1, 2, 3). The total number of FB-positive SChG neurons, their neurochemical characteristics and colocalization patterns are presented in Table 2.

**Table 2:** Neurochemical characteristics of FB-positive neurons supplying porcine intervertebral discs

Immunochemical characteristics					
Groups	FB+/DBH+/NPY+	FB+/DBH+/NPY-	FB+/DBH−/NPY+	FB+/DBH−/NPY-	No. of neurons
CTRL	70.1 ± 2.3	10.3 ± 0.3	5.5 ± 3.0	13.7 ± 3.4	894
E1	18.9 ± 0.7	38.3 ± 0.6	0	42.9 ± 1.2	789
E2	34.6 ± 3.4	41.2 ± 6.0	0	19.8 ± 2.5	844
E3	17.6 ± 1.4	62.5 ± 1.3	0	20.0 ± 1.8	914
	FB+/DBH+/SOM+	FB+/DBH+/SOM-	FB+/DBH−/SOM+	FB+/DBH−/SOM-	
CTRL	17.8 ± 1.0	64.1 ± 0.3	1.1 ± 0.4	16.3 ± 1.0	1154
E1	15.4 ± 1.8	58.1 ± 2.5	2.3 ± 0.2	24.9 ± 1.4	1017
E2	26.1 ± 2.1	41.4 ± 2.1	2.9 ± 1/2	29.8 ± 1.3	919
E3	19.2 ± 3.1	52.0 ± 3.5	2.8 ± 0.3	26.7 ± 0.4	976
	FB+/DBH+/LENK+	FB+/DBH+/LENK-	FB+/DBH−/LENK+	FB+/DBH−/LENK-	
CTRL	15.01 ± 1.7	66.9 ± 2.9	3.9 ± 1.2	14.1 ± 3.2	862
E1	8.8 ± 1.0	58.9 ± 2.7	0	33.7 ± 1.8	741
E2	14.6 ± 4.4	70.0 ± 2.7	0	16.1 ± 2.8	691
E3	4.8 ± 0.8	69.5 ± 3.8	0	25.8 ± 2.9	651
	FB+/DBH+/GAL+	FB+/DBH+/GAL-	FB+/DBH−/GAL+	FB+/DBH−/GAL-	
CTRL	8.4 ± 1.3	72.2 ± 1.3	0	19.0 ± 0.5	816
E1	1.8 ± 0.4	73.5 ± 0.7	0	24.8 ± 1.0	754
E2	0.9 ± 0.3	74.7 ± 4.0	4.2 ± 1.1	17.3 ± 1.9	761
E3	3.2 ± 1.3	74.6 ± 0.5	0	22.8 ± 1.6	698

The total numbers of all examined FB-positive SChG neurons and their colocalisation patterns are given. CTRL – control, E1 – experimental group 1 (two weeks post injection), E2 – experimental group 2 (one month post injection), E3 – experimental group 3 (three months post injection). The results are presented as the mean percentage ± SEM002E

In the control group, the highest number of FB+ neurons was positive for DBH (80% ± 1.5%) and NPY (75.6% ± 2.65%). The number of FB+ labelled for SOM, LENK and GAL was relatively smaller and accounted for 18.6% ± 0.7%, 17.8 ± 1.7%, and 5.5% ± 0.5% of all FB+ neurons, respectively. Similar expression patterns were also observed in the experimental groups; however, there was a difference between the numbers of neurons stained for a given substance between all experimental groups studied.

In experimental group 1, the following staining patterns were observed: DBH (68% ± 1.6%), NPY (18.7% ± 0.5%), SOM (16.8% ± 1.1%), LENK (8.8% ± 0.75%) and GAL (1.9% ± 0.3%). In experimental group 2, the staining patterns were as follows: DBH (77.9% ± 3.1%), NPY (38.4% ± 2.3%), SOM (29.4% ± 1.3%), LENK 15.1% ± 2%) and GAL (4.4% ± 1%). Finally, in experimental group 3, the following staining patterns were observed: DBH (74.2% ± 2.2%), NPY (17.2% ± 0.7%), SOM (17.7% ± 2.4%), LENK (5.6% ± 0.1%), and GAL (3.5% ± 0.6%). In all experimental groups, the greatest decline in immunohistochemical expression was observed for NPY (Figs. 1, 2, 3).

## Discussion

Currently, studies on intervertebral disc sympathetic innervation are very scarce and are mostly limited to rat and human post-mortem studies. Here, for the first time, we established the origins of sympathetic neurons supplying the lumbar intervertebral discs and demonstrated changes in neuropeptide expression over time.

The results of our studies show that the vast majority of lumbar L_4_-L_5_ intervertebral disc sympathetic innervation at the injection site stemmed from SChG neurons localised between L_2_ to L_6_; however, a small amount of FB+ sympathetic neurons was also found further from the injection site, spreading from Th_6_ to S_2_ in all groups of animals.

At the baseline level (control group), DBH, an enzyme catalysing conversion of dopamine into noradrenaline, considered as a classical marker of sympathetic noradrenergic neurons, was the most prevalent molecule studied, observed in 80% of all intervertebral disc neurons. NPY was the most prevalent neuropeptide and was present in 75.6% of all neurons. SOM and LENK were present in 18.6 and 17.5% of all neurons, respectively. Finally, GAL was the least detectable neurochemical and was observed in only 5.5% of all intervertebral disc neurons.

The levels of all but one of the neurochemicals declined over time. The greatest reduction in expression was observed two weeks and two months post injection (E1 and E3 group), and the lowest reduction (DBH, NPY, LENK, GAL) and a significant increase (SOM) in expression were noticed one month post injection (E2 group), probably marking this time point as the best window for any post-injection manipulation, drug delivery and cell and tissue grafting.

Until now, the details of neurochemical dynamics and their characteristics have not been analysed. Most of the available data refer to the neurochemical properties of intervertebral disc sensory innervation without much insight into its autonomic part. Studies by Fujimoto Aoik et al. showed that the most prevalent neurochemical in sensory neurons supplying the lumbar and cervical intervertebral discs is CGRP, which is also present in some subsets of stellate ganglion sympathetic neurons supplying cervical intervertebral discs. Previous studies on intervertebral disc degeneration models have not addressed the expression of other sympathetic nervous system-related molecules, such as DBH, NPY, SOM, LENK, and GAL. Their role in pain transmission in degenerating discs has not been explored in detail; however, recent reports have shown that they are involved in conducting and/or modulating pain that is often manifested in intervertebral disc degeneration as discogenic pain.

The role of the sympathetic noradrenergic innervation in pain transmission and perception has been well established in numerous studies of pain in human patients and animal models (Li et al. 2002; Ma and Eisenach 2003; Wood 2008). The most recent studies by Nascimento et al. have shown that DBH levels are increased in rats after sciatic nerve injury, correlating with mechanical allodynia and pain-like behaviour in these animals. These authors also showed that the chemical suppression of sciatic nerve sympathetic innervation significantly reduced pain perception and decreased sensitivity to cold, thus further supporting the involvement of the noradrenergic system in pain generation (Nascimento et al. 2015).

NPY, which is mainly linked to the regulation of hunger and obesity, has recently been shown to contribute to osteoporosis- and osteoarthritis-related pain generation and perception in postmenopausal women (Xiao et al. 2016), patients with knee osteoarthritis (Wang et al. 2014) and in rat models of this disease (Adaes et al. 2015). Furthermore, studies on NPY expression in animal pain models revealed that NPY was upregulated in the prefrontal cortex and sciatic nerve post-injury, thus further supporting the role of this neuropeptide role in pain generation and perception (Kimura et al. 2015; Magnussen et al. 2015). In addition, recent studies of sensory neurons supplying intervertebral discs in small animal disc degeneration models demonstrated that the expression of NPY is increased, suggesting an enhanced nociceptive response and a contribution to discogenic pain (Miyagi et al. 2014).

SOM is another neuropeptide whose association with pain manifestation has been gradually established over recent years (Kumar 2009). For example, a study by Carlton et al. investigating the role of SOM receptors in analgesia showed that SOM inhibits activity-dependent │ nociceptors, alleviating pain and reducing pain perception in patients following intrathecal or epidural administration (Carlton et al. 2001). More importantly, SOM has been indicated as a plausible alternative to other dependency-evoking analgesics for alleviating pain during/post minor surgery procedures and for controlling different forms of pain signals induced by certain conditions and disorders, such as joint inflammation, arthritis, and neuropathy (Abdel-Magid 2015; Carlton et al. 2001; Silveri et al. 1994).

LENK belongs to a well-established family of endogenous opioids, which are naturally occurring painkillers. Recent studies have shown that the stimulation of endogenous LENK production, the administration of exogenous LENK or blocking the activity of endogenous LENK-degrading enzymes affects pain signalling and manifestation by treating chronic pain of various origins (Goins et al. 2012), alleviating visceral pain in mouse models of painful bladder syndrome (Yokoyama et al. 2009), reducing post-injury neuropathic pain in mice (Bonnard et al. 2016), and promoting peripheral opioid analgesia in rats (Akahori et al. 2008; Schreiter et al. 2012). Furthermore, the results of clinical trials on the use of LENK analogues have revealed that the administration of exogenous LENK helps to lessen chronic pain in cancer patients (Moulin et al. 1985), thus underlining the importance of LENK in pain generation and treatment.

Similarly, GAL is yet another example of neuropeptides involved in pain signalling, acting in the suppression of nociceptive receptors and contributing to pain alleviation in animal models of chronic and acute pain (Coronel et al. 2017; Liu and Hokfelt 2002). Extensive studies on the role of GAL in pain transduction and the roles of its agonists and antagonists have been conducted for over 30 years and have presented new evidence for the importance of this neuropeptide in pain modulation and highlighted its role as an effective and less side effect-inducing painkiller candidate of the next generation of analgesics used to treat different forms of pain (Lang et al. 2015). It has been shown that the effects of GAL on pain signalling are dose dependent, either alleviating or increasing pain perception in the studied animal pain models (Holmes et al. 2003).

Here, we observed that at the 1-month time point, which was established as a key time point for future experiments, the three studied peptided, i.e., DBH, NPY and GAL, that are directly or indirectly involved in pain generation and its increased manifestation were downregulated in all studied groups. By contrast, the expression levels of SOM and LENK, the two neuropeptides linked to pain suppression and modulation, were increased or remained at a similar level to baseline, respectively. The observed findings indicate that intervertebral disc manipulations trigger detectable changes in the neurochemical complexity of disc sympathetic innervation and likely contribute to altered pain sensation at different time points following clinical intervention. It may be concluded that modulating neuropeptide levels could help to alleviate the pain induced by intervertebral disc degeneration, providing a better outcome for degenerating disc therapy and improving overall prognosis.

It must be noted, however, that our study has certain limitations resulting from its observational nature. Due to ethical and technical considerations, neither actual pain nor the morphology of the injured degenerating disc was measured.

Further studies investigating the complexity of the dynamics of neuropeptides in experimentally induced disc compression and degeneration in pigs have been designed and are likely to provide more answers concerning the role that these neurochemicals play in pain modulation and perception, ultimately contributing to a better understanding of pain signalling in intervertebral disc diseases.
